# CT radiomics and human-machine hybrid system for differentiating mediastinal lymphomas from thymic epithelial tumors

**DOI:** 10.1186/s40644-024-00808-2

**Published:** 2024-11-28

**Authors:** Han Xia, Jiahui Yu, Kehui Nie, Jun Yang, Li Zhu, Shengjian Zhang

**Affiliations:** 1 Department of Radiology, Fudan University Shanghai Cancer Center and Department of Oncology, Shanghai Medical College, Fudan University, No. 270, Dongan Rd, Shanghai, 200032 People’s Republic of China; 2 Department of Radiology, Shanghai Chest Hospital, School of Medicine, Shanghai Jiao Tong University, No. 241, West Huaihai Rd, Shanghai, 200030 People’s Republic of China; 3 Taimei Medical Technology Co., Ltd, Shanghai, 200032 People’s Republic of China

**Keywords:** Lymphoma, Thymic epithelial tumors, Computed tomography, Radiomics, Ensemble learning

## Abstract

**Background:**

It is difficult for radiologists, especially junior radiologists with limited experience to make differential diagnoses between mediastinal lymphomas and thymic epithelial tumors (TETs) due to the overlapping imaging features. The purpose of this study was to develop and validate a CT-based clinico-radiomics model for differentiating lymphomas from TETs and to investigate whether a human-machine hybrid system can assist junior radiologists in improving their diagnostic performance.

**Methods:**

The patients who underwent contrast-enhanced chest CT and pathologically confirmed with lymphoma or TET at two centers from January 2011 to December 2019 and from January 2017 to December 2021 were retrospectively included and split as training/validation set and external test set, respectively. Clinical and radiomic signatures were pre-selected by elastic-net, and the models were established with the selected signatures using ensemble learning. Three radiologists independently reviewed CT images and assessed each case of the external test set with knowledge of the relevant clinical information. The diagnoses of reader 1, reader 2, and reader 3 were compared with those of the models in the external test set and further separately input to the model’s ensemble process as a human-machine system to make final decisions in the external test set. The improvement of diagnostic performance of radiologists by human-machine system was evaluated by the area under the receiver operating characteristic curve and increase rate.

**Results:**

A total of 95 patients (51 with lymphomas and 44 with TETs) at Center 1 and 94 (52 with lymphomas and 42 with TETs) at Center 2 were enrolled and divided into training/validation sets and external test set, respectively. The diagnostic performance of the clinico-radiomics model has outperformed the junior radiologists and senior radiologist in AUC (clinico-radiomics model: 0.85 (0.76,0.92); reader 2: 0.70 (0.60,0.80); reader 3: 0.60 (0.49,0.71), reader 1: 0.76 (0.66,0.86), respectively) in the external test set. The human-machine hybrid system demonstrated significant increases in AUC (reader 1 + model: 0.87 (0.79,0.94), an increase of 14%; reader 2 + model: 0.86 (0.77,0.93), an increase of 23%; reader 3 + model: 0.84 (0.76,0.91), an increase of 40%), compared to the human performance alone.

**Conclusions:**

The clinico-radiomics model outperformed three radiologists in differentiating lymphomas from TETs on CT. The use of the human-machine hybrid system significantly improved the performance of radiologists, especially junior radiologists. It provides a real-time decision tool to reduce bias and mistakes in radiologist diagnosis and enhances the diagnostic confidence of junior radiologists. This attempt may lead to more human-machine hybrid systems being explored in the diagnosis of different diseases to drive future clinical applications.

**Supplementary Information:**

The online version contains supplementary material available at 10.1186/s40644-024-00808-2.

## Background

Thymic epithelial tumors (TETs), including thymic carcinomas and thymomas, and lymphomas are the two most common tumors in the mediastinum, accounting for about 28% and 16% of mediastinal tumors, respectively [1]. There are different therapeutic strategies for them: treatment of TETs is mainly based on surgery combined with chemoradiotherapy [2], while lymphoma is mainly treated with chemotherapy and radiotherapy [3, 4]. However, given that there is a high rate of non-therapeutic thymectomies currently, of which mediastinal lymphomas consist of 54.3% of non-therapeutic thymectomies [5, 6], the differential diagnosis of these two tumors is of great clinical significance. Although several studies have evaluated the different imaging findings between TETs and lymphomas, there are still some features overlapping between the two, especially between Hodgkin lymphoma or certain types of non-Hodgkin lymphoma such as large B-cell lymphoma and TETs, leading to misdiagnosis and non-therapeutic thymectomies [6–9]. Biopsy is considered as the reference standard for pretreatment assessment but suffers from the disadvantages of sampling errors [10, 11] and the risk of metastasis [12], thus it is necessary to develop non-invasive methods for accurate pretreatment diagnosis.

Several studies investigated the ability of PET, MRI, and other functional imaging to distinguish TETs from lymphomas [13–19]. However, according to the 2023 NCCN guidelines for thymomas and thymic carcinomas, imaging evaluation of mediastinal masses before treatment with contrast-enhanced CT remains preferred [2], and CT is the primary method for chest imaging, thus it is more clinically significant to improve the ability to distinguish TETs from lymphomas on CT.

Radiomics can reflect the biological heterogeneity of tumor lesions by extracting image features that cannot be directly interpreted by human eyes. The assessment of tumor heterogeneity by radiomics has become a non-invasive tool for diagnosis, prognosis, and treatment response in different diseases, such as neuroendocrine carcinoma, colorectal cancer, and lung cancer [20–22]. Given that lymphoma is a hypercellular tumor and has fewer collagen fibers and micro-necrosis than TETs [23, 24], a CT-based radiomics model may be helpful for differential diagnosis. A few single-center studies have investigated the role of radiomics-based models in the diagnosis of mediastinal lesions and achieved good performance [25–27]. However, no one assessed the generalizability of models in the external test set and investigated the assistive value of radiomics for junior radiologists who are inexperienced with differential diagnosis of TETs and lymphomas.

This study aimed to develop and validate a CT-based clinico-radiomics model for differentiating lymphomas from TETs and to investigate whether a human-machine hybrid system (reader + clinico-radiomics model) can assist junior radiologists in improving their diagnostic performance to reduce subjective bias and help them to improve their clinical judgment.

## Patients and Methods

The institutional review board from each involved center approved this retrospective study, and the informed consent was waived.

### Patient cohort

**Fig. 1:**
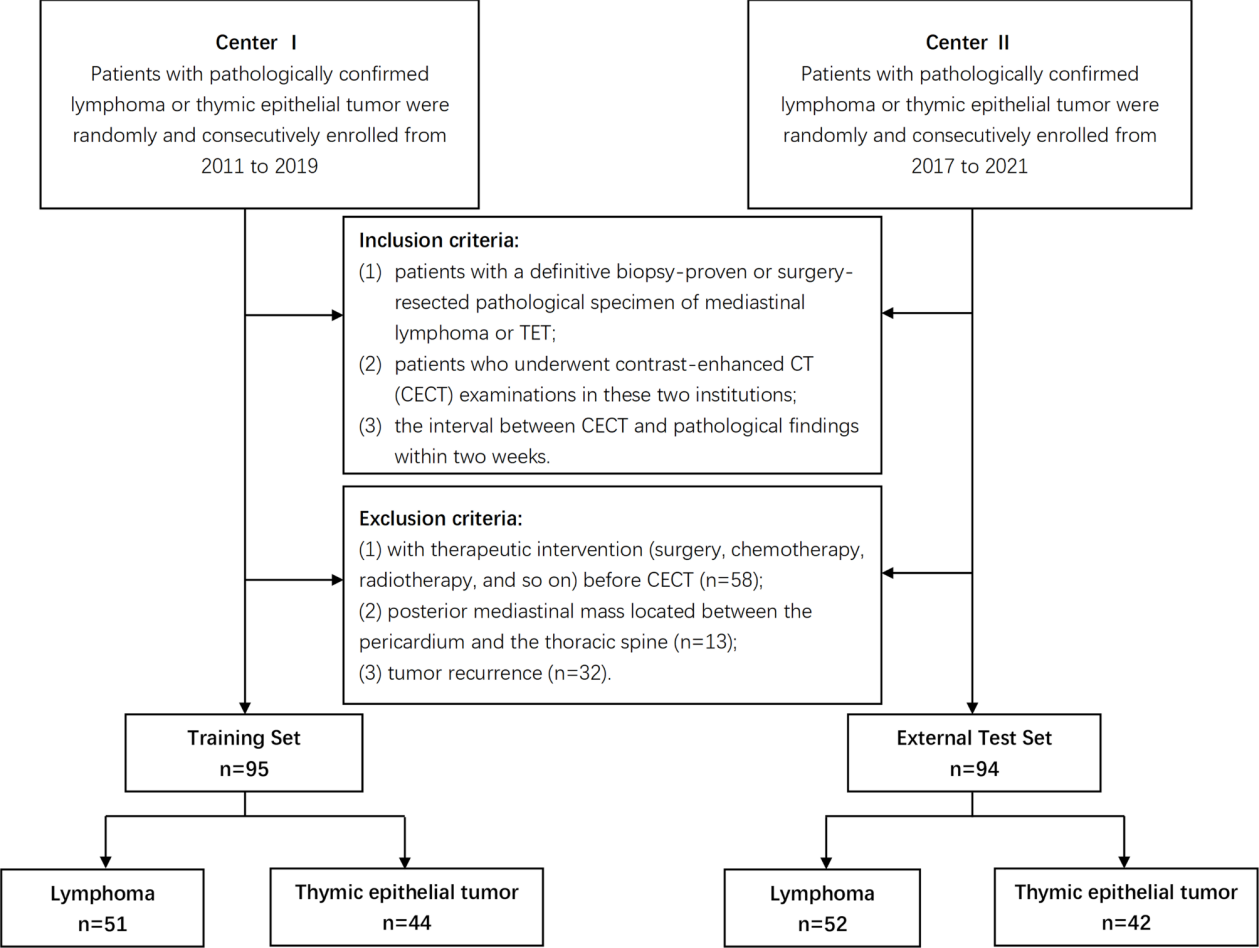
Flowchart of patient selection. TET, thymic epithelial tumor

Patients with a histological diagnosis of lymphoma or TET from two medical centers (Center 1: Shanghai Cancer Center, Shanghai, China; Center 2: Shanghai Chest Hospital, Shanghai, China) between January 2011 and December 2019 and between January 2017 and December 2021 were consecutively reviewed. Two radiologists (H.X. and J.H.Y) independently screened cases according to the predefined criteria. The inclusion criteria were (1) patients with a definitive biopsy-proven or surgery-resected pathological specimen of mediastinal lymphoma or TET; (2) patients who underwent contrast-enhanced CT (CECT) examinations in these two institutions; and (3) the interval between CECT and pathological findings within two weeks. A total of 292 patients were identified. The exclusion criteria were (1) with therapeutic intervention (surgery, chemotherapy, radiotherapy, and so on) before CECT (*n* = 58); (2) posterior mediastinal mass located between the pericardium and the thoracic spine (*n* = 13); and (3) tumor recurrence (*n* = 32). The patients enrolled at Center 1 and Center 2 were assigned to the training set and external test set, respectively. A flowchart of patient selection in the study is shown in Fig. 1.

### Clinicopathological data

The demographic characteristics and baseline clinical data were collected from the medical records and laboratory reports, including sex, age, B symptoms, autoimmune disease, chest pain, respiratory symptoms, white blood cell count, lymphocyte count, lactate dehydrogenase (LDH), and therapeutic strategies. Histopathological data of all patients were extracted from the pathologic reports in the electronic medical records system.

### CECT image acquisition

All CECT images were obtained using a 40-slice multidetector CT scanner (SOMATOM Sensation 40; Siemens Healthineers) or 64-detector row CT scanners (SOMATOM Sensation 64; Siemens Healthineers or Brilliance 64, Philips Medical Systems) at center 1 and 64-slice CT scanners (Discovery CT750 HD; GE Healthcare or Brilliance 64, Philips Medical Systems) or 256-detector row scanners (Brilliance iCT; Philips Medical Systems or Revolution CT, GE Healthcare) at center 2. A total of 80-100mL (1.5mL/kg) iodinated contrast agent was injected via the antecubital vein at a flow rate of 2–3 mL/s for each enrolled subject. CECT scans were initiated at 40s after injection of the contrast agent. The scanning protocol parameters were set as follows: 120 kVp, 100–300 mAs or auto-mAs, 5 mm section interval, 5 mm slice thickness, and a field of view 350–500 mm.

### Lesion segmentation and radiomic feature extraction

Axial CECT images were loaded into 3D Slicer software (version 5.0.3) for lesion segmentation. A radiologist (H.X., a chest radiology resident with two years of experience) manually delineated volumes of interest (VOIs) on the axial CT slices covering the lesions. The anonymous VOIs were reviewed and adjusted if needed by a senior radiologist (S.J. Z) with more than 20 years of experience in chest CT for quality assurance.

Radiomic features were extracted using the LIFEx package (version 7.2.0) [28]. CECT images were resampled to 1 × 1 × 1 mm³ voxels by use of three-dimensional Lagrangian polygon interpolation. The grey-level intensity of the image was discretized at a fixed bin width of 10 Hounsfield units (HU) within a range from − 1000 to 3000 HU. For each case, we calculated a total of 117 quantitative features from images, including 14 morphological characteristics, 47 first-order features derived from histogram analysis, and 56 second-order features calculated from four grey-level matrices (Gray Level Co-occurrence Matrix, Gray Level Run-Length Matrix, Neighboring Gray Tone Difference Matrix, Gray Level Size Zone Matrix). Values of all the extracted radiomic features were standardized using the Z-score method.

### Feature selection and radiomics model development

The data from Center 1 were split into training and validation subsets at a ratio of 7:3. Feature selection and model development were performed on the training data set. A linear elastic-net model was used for feature selection, and the regularization and penalty coefficients of elastic-net were tuned by a five-fold cross-validation and grid search. Based on the selected clinical and radiomics features, the clinico-radiomics model was developed as an integration of three pre-trained classification algorithms, support vector machine (SVM), Bayes, and logistic regression (i.e., named as ensemble classifier). In this ensemble classifier, the predicted probabilities from each of the three pre-trained classification algorithms were generated and averaged by soft voting, which produced final predictions. A soft voting classifier bases on probabilistic values generated by several classification algorithms, and the voting result is a weighted average of probabilities of all pre-trained algorithms in predicting certain classifications.

Additionally, a model with only radiomics signatures (radiomics-only model) was built using the training set. Feature selection and model training methods were the same as that for the clinico-radiomics model, except that no clinical variables were included for model inputs. All the developed models were validated and fine-tuned with the internal validation data from Center 1.

### External validation for the radiomics-based models and human-machine hybrid models versus readers

All trained models were tested using the external data set from Center 2. To compare the performance of human readers to the models, three radiologists, one senior reader (S.J. Z, with more than 20 years of experience in chest radiology, reader 1), and two junior readers (L.Z. and H.L.W, with 2- and 4.5 years of experiences in chest radiology, respectively, reader 2 and reader 3) independently reviewed all CECT images and relevant clinical information (such as age, symptoms and LDH) for each case in the external test set and made their own diagnoses in differentiating lesions as lymphoma or TET, completely blinded to the pathology.

**Fig. 2:**
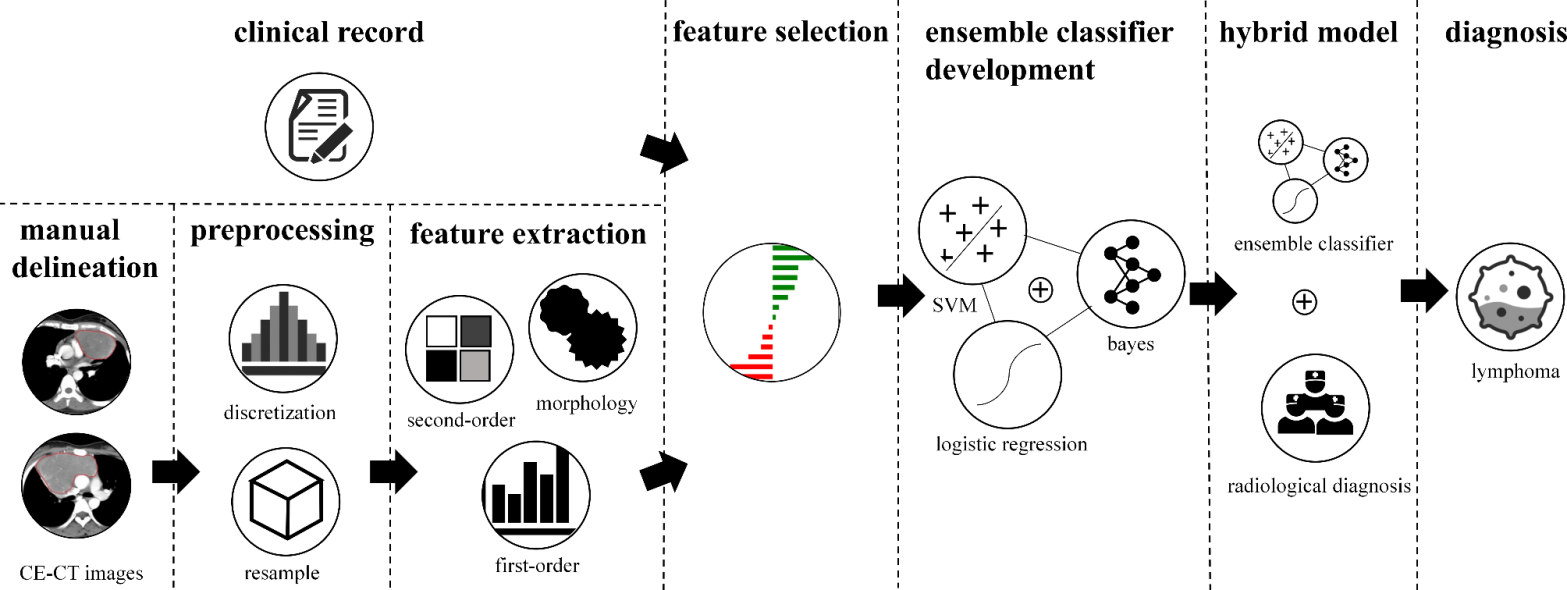
Schema of the human-machine hybrid system development. The ensemble classifier was developed as an integration of three pre-trained classification algorithms, support vector machine (SVM), Bayes, and logistic regression. In this ensemble classifier, the predicted probabilities from each of the three pre-trained classification algorithms were generated and averaged by soft voting, which produced final predictions. The hybrid models were developed by integrating the diagnoses of each radiologist into the ensemble classifier’s voting process to derive the final results

We further explored whether the combination of machine-extracted features and human readings (a human-machine hybrid system) would improve the performance of radiologists, and provide an additive benefit on diagnostic accuracy in clinical practice. The diagnoses of each radiologist were integrated into the clinico-radiomics model’s voting process to derive the final results in human-machine hybrid systems (reader 1 + model; reader 2 + model; reader 3 + model). The overall workflow of the human-machine hybrid system development is illustrated in Fig. 2.

### Statistical analysis

Qualitative variables were presented as frequencies, continuous variables were shown as the median and quartiles. The clinical characteristics were compared using the Mann-Whitney U test for continuous variables and using chi-square test or Fisher’s exact test for categorical variables between lymphomas and TETs. The sensitivity, specificity, accuracy, and AUC of the models and performance of the three readers in the internal validation set and external test set were calculated. All statistical analyses and model development/validation were performed with R (version 4.2.1) and Python (version 3.8.0). The p-value < 0.05 was set as statistical significance.

## Results

### Clinical characteristics in Center 1 and Center 2

A total of 189 patients were included in the study. Clinical characteristics of patients from Center 1 (*n* = 95) and Center 2 (*n* = 94) were summarized in Table 1. The pathological subtypes and treatments of lymphomas and TETs were also shown. In Center 1, fifty-one patients were diagnosed with lymphomas, and forty-four patients were with TETs. In Center 2, fifty-two patients were diagnosed with lymphomas and forty-two patients with TETs. The lymphoma patients showed younger age (30 [24.5–37.5]vs 51.5 [45-59.5] and 35 [29.8–41.3] vs. 52[35–62], both *p* < .001), fewer autoimmune diseases (1.9% vs. 20.5%, *p* = .01 and 0% vs. 16.7%, *p* = .008), higher frequency of respiratory symptoms (76.5% vs. 34.1% and 32.7% vs. 2.4%, both *p* < .001), lower lymphocyte count (1.2 [0.9–1.5] vs. 1.8 [1.5–2.4], *p* < .001 and 1.3 [0.9–1.5] vs. 1.5 [1.1–1.9], *p* = .2), and higher LDH level (277 [187–432] vs. 176 [153–199], *p* < .001 and 233 [185–434] vs. 184 [164–224], *p* = .001) than patients with TETs in both centers, while no significant difference was found in sex (47.1% vs. 54.5%, *p* = .60 and 30.8% vs. 52.4%, *p* = .06), chest pain (19.6% vs. 15.9%, *p* = .84 and 7.7% vs. 2.4%, *p* = .497), and white blood cell count (6.7 [5.8–8.5] vs. 6.2 [5.4–7.4], *p* = .12 and 7.3 [5.5-9.0] vs. 6.4 [5.3–7.7], *p* = .22) between lymphomas and TETs.

**Table 1:** Patient demographics and clinical characteristics in Center 1 and Center 2

Variables	Training set (*n* = 95)		P-value	External test set (*n* = 94)		P-value
	Lymphoma (*n* = 51)	TETs (*n* = 44)		Lymphoma (*n* = 52)	TETs (*n* = 42)	
Sex(M/F)	24/27	24/20	0.602	16/36	22/20	0.056
Age, year	30 (24.5–37.5)	51.5 (45-59.5)	< 0.001*	35 (29.8–41.3)	52 (35–62)	< 0.001*
Thymoma	-	37		-	42	
Thymic carcinoma	-	7		-	0	
Primary mediastinal large B-cell lymphoma	45	-		30	-	
Hodgkin disease	3	-		22	-	
Mucosa-associated lymphoid tissue	2	-		0	-	
T-lymphoblastic lymphoma	1	-		0	-	
B symptom (Yes/No)	17/34	0/44	< 0.001*	2/50	0/42	0.500
Autoimmune disease (Yes/No)	1/50	9/35	0.010*	0/52	7/35	0.008*
Chest pain (Yes/No)	10/41	7/37	0.841	4/48	1/41	0.497
Respiratory symptoms (Yes/No)	39/12	15/29	< 0.001*	17/35	1/41	< 0.001*
White blood cell count (×10^9^/L)	6.7 (5.8–8.5)	6.2 (5.4–7.4)	0.117	7.3 (5.5-9.0)	6.4 (5.3–7.7)	0.220
Lymphocyte count (×10^9^/L)	1.2 (0.9–1.5)	1.8 (1.5–2.4)	< 0.001*	1.3 (0.9–1.5)	1.5 (1.1–1.9)	0.016*
Lactate dehydrogenase (IU/L)	277 (187–432)	176 (153–199)	< 0.001*	233 (185–434)	184 (164–224)	0.001*
Chemotherapy	42	4	< 0.001*	1	2	< 0.001*
Surgery	7	39		24	40	
None	2	1		27	0	

An asterisk superscript (*) indicated that the p-value less than 0.05

### Important radiomic and clinical features

**Fig. 3:**
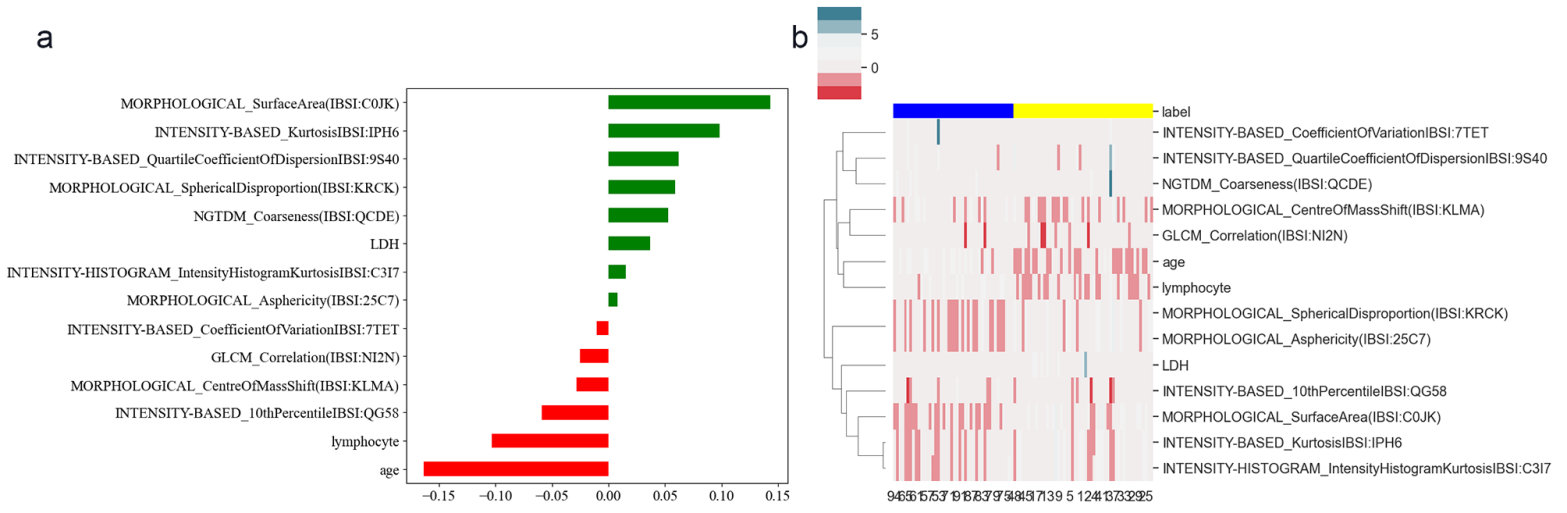
Selected important features in the training data set. (**a**) The fourteen important features selected by elastic-net. The extent of feature importance was defined by the width of the bars. Negative regression coefficients were shown in red, and positive coefficients were shown in green. (**b**) Cluster heatmap of these selected features for differentiating lymphomas from TETs. TETs, thymic epithelial tumors

For the clinico-radiomics model, the fourteen most relevant features were selected, age, LDH, lymphocyte count, four morphology-based features (Surface Area, Spherical Disproportion, Asphericity, and Centre of MassShift), five intensity-based features (Kurosis, Quartile Coefficient of Dispersion, Intensity Histogram Kurtosis, Coefficient of Variation, and 10th Percentile), one GLCM feature (Correlation) and one NGTDM feature (Coarseness). Feature importance was ranked as indicated in Fig. 3(a), the top five features were age (regression coefficient, rc= -0.16), morphological-based surface area (rc = 0.14), lymphocyte (rc= -0.1), intensity-based kurtosis (rc = 0.1) and quartile coefficient of dispersion (rc = 0.06). The differentiations of the selected features between the lymphoma and TET groups were illustrated by a heat map in Fig. 3(b).

### Performance of radiologists and radiomics models

For the training data, the clinico-radiomics model achieved a sensitivity of 94% and, a specificity of 100% with an AUC of 1.00. For the internal validation set, the radiomics-only model reached an AUC, sensitivity, specificity, and accuracy of 0.86, 80%, 67%, and 75%, respectively. The AUC of the clinico-radiomics model was 0.86, and the sensitivity, specificity, and accuracy were 75%, 89%, and 79%, respectively.

**Fig. 4:**
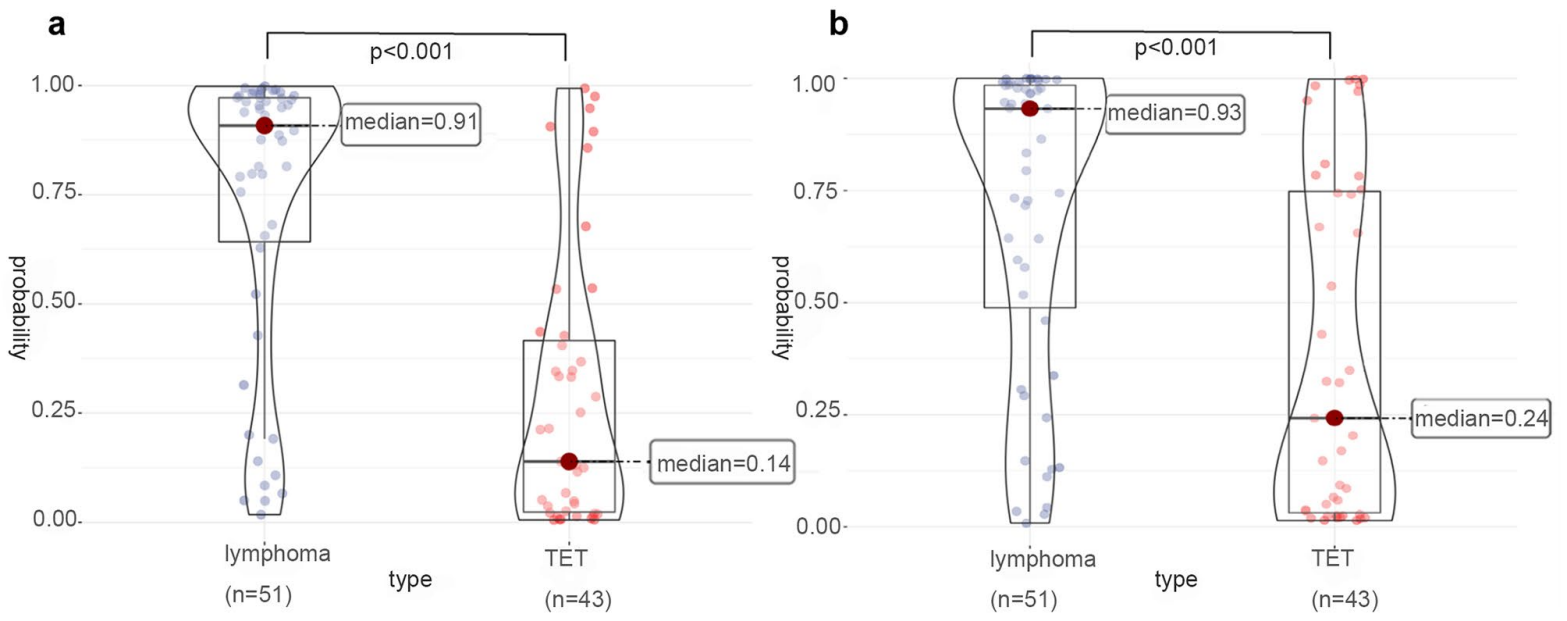
The predicted probability of lymphomas and TETs in the external test set. (**a**) Distribution of predictive probabilities of radiomic-only model for lymphoma and TET. The median predicted probability for lymphoma was 0.91, and the median predicted probability for TET was 0.14. There was a significant difference in probability between the two (*p* < .001). (**b**) Distribution of predictive probabilities of clinico-radiomics model for lymphoma and TET. The median predicted probability for lymphoma was 0.93, and the median predicted probability for TET was 0.24. There was a significant difference in probability between the two (*p* < .001)

**Fig. 5:**
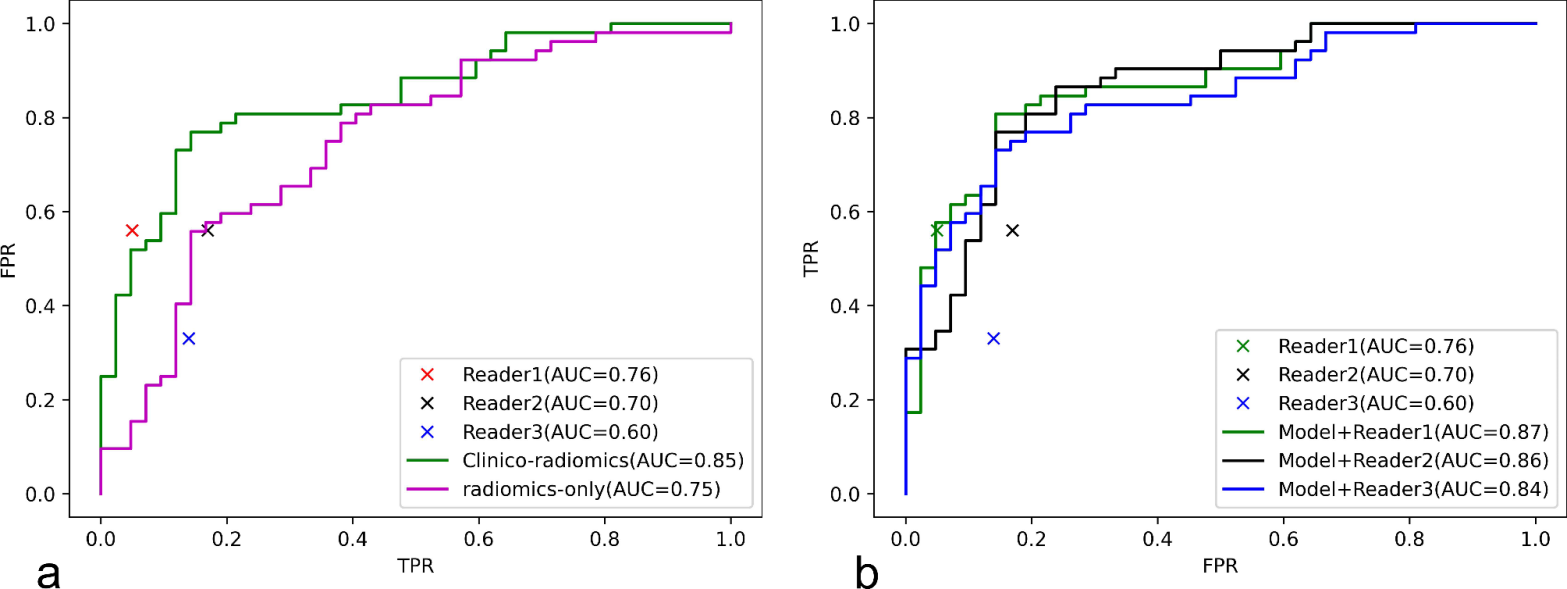
Diagnostic performance of models and three radiologists. (**a**) The ROC curves of the models versus three readers (senior radiologist: reader 1; junior radiologist: reader 2 and 3) for differentiating lymphomas from TETs in the external test set. (**b**) Performance comparison of three readers versus human-machine hybrid models in the external test set. The human-machine hybrid models improved the AUC from 0.76 to 0.87 for reader 1, improved the AUC from 0.70 to 0.86 for reader 2, and improved the AUC from 0.60 to 0.84 for reader 3

In the external test set, the performances of the radiomics-only model and clinico-radiomics model were compared to that of radiologists. The AUC, sensitivity, specificity, and accuracy of the radiomics-only model were 0.75, 75%, 64%, and 70%. The AUC of the clinico-radiomics model was 0.85, and the sensitivity, specificity, and accuracy were 79%, 81%, and 80%, respectively (Table 2). The predicted probabilities of the radiomics-only model and clinico-radiomics model were visualized on violin plots in the external test set (Fig. 4). Compared to that of radiologists, the AUC, sensitivity, specificity, and accuracy were 0.76, 56%,95%,73% for reader 1, and 0.70, 56%, 83%, 68% for reader 2, and 0.60, 33%, 86%, 56% for reader 3. The discriminative power of the radiomics-only model was comparable to those of the radiologists and the clinico-radiomics model was superior to the performance of the radiologists. The ROC curve comparisons of models to radiologists are shown in Fig. 5(a).

**Table 2:** Diagnostic performance of the models and three readers in the external test set

Metric	Radiomics-only model	Clinico-radiomics model	Reader 1	Reader 2	Reader 3
sensitivity	0.75(0.66,0.83)	0.79(0.66,0.88)	0.56(0.44,0.67)	0.56(0.44,0.68)	0.33(0.24,0.42)
specificity	0.64(0.54,0.73)	0.81(0.67,0.90)	0.95(0.86,1)	0.83(0.70,0.96)	0.86(0.79,0.93)
accuracy	0.70(0.60,0.79)	0.80(0.72,0.88)	0.73(0.63,0.82)	0.68(0.59,0.77)	0.56(0.46,0.66)
AUC	0.75(0.68,0.82)	0.85(0.76,0.92)	0.76(0.66,0.86)	0.70(0.60,0.80)	0.60(0.49,0.71)

### Performance of the human-machine hybrid system and human reading improvement

In this study, we integrated the diagnoses of radiologists within the voting process of the clinico-radiomics model to make final decisions, which was named a human-machine hybrid system (human readings combined with the clinico-radiomics model). For reader 1, the AUC increased from 0.76 to 0.87 (an increase of 14%). For reader 2, the AUC increased from 0.70 to 0.86 (an increase of 23%). For reader 3, the AUC increased from 0.60 to 0.84 (an increase of 40%). The performance metrics are summarized in Table 3. The ROC-AUCs of the human-machine hybrid systems (R1 + model; R2 + model; R3 + model) and three radiologists were illustrated in Fig. 5(b).

**Table 3:** Performance of the hybrid models and three readers in the external test set

Metric	R1	R1 + model	R2	R2 + model	R3	R3 + model
Sensitivity	0.56(0.44,0.67)	0.81(0.68,0.89)	0.56(0.44,0.68)	0.87(0.75,0.93)	0.33(0.24,0.42)	0.73(0.60,0.83)
Specificity	0.95(0.86,1)	0.86(0.72,0.93)	0.83(0.70,0.96)	0.76(0.61,0.87)	0.86(0.79,0.93)	0.86(0.72,0.93)
Accuracy	0.73(0.63,0.82)	0.83(0.75,0.91)	0.68(0.59,0.77)	0.82(0.74,0.90)	0.56(0.46,0.66)	0.79(0.70,0.87)
AUC	0.76(0.66,0.86)	0.87(0.79,0.94)	0.70(0.60,0.80)	0.86(0.77,0.93)	0.60(0.49,0.71)	0.84(0.76,0.91)

* R1, R2 and R3 represented reader1 (senior reader), reader 2 and reader 3 (junior radiologists), respectively. The clinico-radiomics model combined with readers represented the hybrid models (R1 + model, R2 + model and R3 + model).

## Discussion

In this proof-of-concept study, a machine learning-based model that incorporated clinical factors and CT-based radiomic signature, a clinico-radiomics model, had reached a good performance for differentiating anterior mediastinal lymphomas from TETs, when tested with the internal and external validation sets. The diagnostic performance of the clinico-radiomics model outperformed three radiologists in the external test set. Furthermore, for the first time, we have demonstrated that a human-machine hybrid system could improve the diagnostic performance of the radiologists in differentiating lymphomas from TETs, which is more meaningful since computerized intelligence models aim to help radiologists, instead of replacing and competing with them, especially junior radiologists when they lack diagnostic confidence.

Previous radiomics studies have gained some promising points of radiomics signatures in differentiating solid mediastinal masses from cysts [29], predicting TET grade [30–37], and distinguishing TETs from mediastinal lymphomas [25–27]. Two similar studies [25, 26] used non-enhanced CT-based radiomics models or enhanced CT-based radiomics models to differentiate thymic neoplasms from lymphomas, and the performance AUCs reached over 0.9 even close to 1 in their training and internal validation set. However, these studies were not validated with external test sets, thus their high performance might be due to the overfitting of the models or statistical bias from the data set, which might not be reliable. Compared with the study by He et al. [26], although our study had a higher AUC in the training set and a lower AUC in the internal validation set, the clinico-radiomics model in our study was evaluated using an external data set and the almost identical AUCs (0.86 and 0.85 in the internal validation set and external test set, respectively) indicated the robustness of the model. In addition, we also compared model outputs to the diagnoses of radiologists who had different diagnostic experiences to see the performance level that the models could reach. The radiomics-only model had comparable performance to three radiologists and the clinico-radiomics model had a better discrimination power than that of three readers. Besides, in the external test set, all three radiologists had less confidence and lower sensitivity in diagnosing lymphomas, as compared to diagnoses of TETs, while the clinico-radiomics model reached a balance between the two tumor classes, suggesting a better performance in both sensitivity and specificity.

The hybridization of human and artificial intelligence (AI) emerged as a new form of human-machine collaboration to enhance each other and bring out the greatest potential of both [38]. AI can provide support for human decision-making, or humans can assist the machine learning process to support AI tasks. In our study, combining machine intelligence with human expertise is an appropriate way to maximize the potential of advanced technologies, and with the help of the machine, human readers can perform better diagnoses. The AUC value of the human-machine hybrid systems increased by 14% for reader 1, 23% for reader 2, and 40% for reader 3. The human-machine hybrid system showed improvements in differentiating TETs from lymphomas on CECT, especially for junior radiologists whose experiences were insufficient. The human-machine hybrid system in this study proved this point that a precise diagnosis could be made in the external test data, and the performance was better than radiologists alone. As mentioned above, it can be explained that human diagnoses are influenced by their experience and subjective bias, which can be eliminated by the model to some extent. Moreover, this system could improve the diagnostic skills of junior radiologists by helping them to think and provide real-time feedback. There are limited studies on human-machine hybrid systems [39, 40] and our study made a preliminary attempt. By far as we have acknowledged, this is the first study in combining machine-derived features with human readings for optimizing differential diagnoses of TETs from lymphomas.

There were some limitations in our study. Firstly, the study design was retrospective, the nature of this study might introduce selection bias. Secondly, this study had a relatively small sample size, which might influence the robustness and generalizability of the findings. Thirdly, although this study included two institutions, it was conducted in a single geographical area which might limit the applicability of the findings to broader populations. Moreover, the evaluation of some important features such as lymphadenopathy was not considered, although it could be reflected by the diagnoses of radiologists to some extent. Future exploration with a larger sample size and more radiologists involved from multiple centers is needed.

## Conclusion

In conclusion, the study demonstrated that our clinico-radiomics model which integrated clinical factors with radiomics features of enhanced CT images, could better differentiate lymphomas from TETs than radiologists. The proposed human-machine hybrid system based on radiologist plus machine intelligence was effective in aiding young radiology residents in achieving improved reading accuracy. It provides a real-time decision tool to reduce bias and mistakes in radiologist diagnosis and enhances the diagnostic confidence of junior radiologists. This attempt may lead to more human-machine hybrid systems being explored in the diagnosis of different diseases and the generalizability and translational research of the human-machine hybrid system will also be further investigated in the clinical assessments with a larger subject pool.

## Electronic supplementary material

Below is the link to the electronic supplementary material.

Supplementary Material 1

## Data Availability

The datasets used and /or analyzed during the current study are available from the corresponding author on reasonable request.

## Abbreviations

AUC: Area under the curve
CECT: Contrast-enhanced CT
LDH: Lactate dehydrogenase
VOI: Volume of interest
HU: Hounsfield units
SVM: Support vector machine
GLCM: Gray-level co-occurrence matrix
NGTDM: Neighboring Gray Tone Difference Matrix
ROC: Receiver operating characteristic
AI: Artificial intelligence
