# Supplementary material for: CT radiomics and human-machine hybrid system for differentiating mediastinal lymphomas from thymic epithelial tumors
Source: Cancer Imaging. 2024 Nov 28;24:163. doi: 10.1186/s40644-024-00808-2 (PMC11603948; doi:10.1186/s40644-024-00808-2)
Supplement: Supplementary file 1 — Supplementary Material 1 [file 40644_2024_808_MOESM1_ESM.docx]

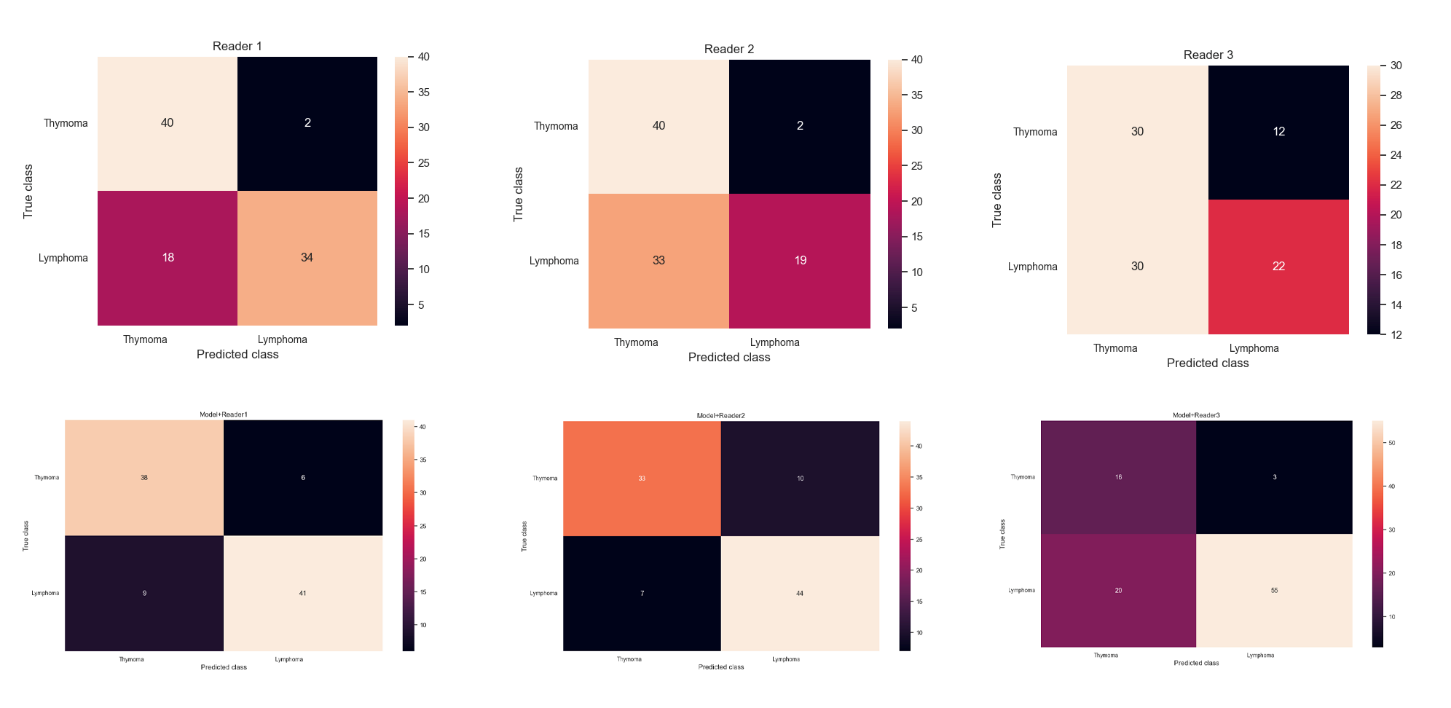
Supplementary materials

**Figure S1.** The confusion matrixes of radiologists and human-machine hybrid systems

McNemar’s test showed that there was no statistical significance between Reader 1 and Reader 1+model of accuracies (p= 0.157). However, there are significant statistical differences between Reader 2 and Reader 2+model (p= 0.021) as well as Reader 3 and Reader 3+model (p= 0.020) of accuracies.
